# Bayesian parametric estimation shows the effects of social prestige and bilingualism on reshaping language competition

**DOI:** 10.1098/rspb.2025.1451

**Published:** 2025-10-29

**Authors:** Sizhe Yang, Tingting Ye, Shengbo Bi, Li Jin, Yongyan Zheng, Menghan Zhang

**Affiliations:** ^1^ Research Institute of Intelligent Complex Systems, Fudan University, Shanghai, People’s Republic of China; ^2^ Ministry of Education Key Laboratory of Contemporary Anthropology, School of Life Sciences, Fudan University, Shanghai, People’s Republic of China; ^3^ State Key Laboratory of Genetics and Development of Complex Phenotypes, Center for Evolutionary Biology, Human Phenome Institute, Zhangjiang Fudan International Innovation Center, School of Life Sciences, Fudan University, Shanghai, People’s Republic of China; ^4^ College of Foreign Languages and Literature, Fudan University, Shanghai, People’s Republic of China; ^5^ Institute of Modern Languages and Linguistics, Fudan University, Shanghai, People’s Republic of China

**Keywords:** language competition model, Bayesian parametric estimation, bilingualism and language prestige, language extinction, culture sustainability

## Abstract

Understanding the mechanisms of language competition is crucial for mitigating language extinction and promoting cultural sustainability. Nevertheless, how to quantify the effects of socio-linguistic factors, such as social prestige and bilingualism, on language competition remains a critical challenge. Here, we present Markov-process-based language competition models to explore the interactions among monolingual and bilingual groups. Based on these models, we develop a Bayesian parametric estimation strategy, which enables quantifying the effects of socio-linguistic factors through rigorous statistical examinations. With six empirical cases worldwide, we observe a general trend of minority monolinguals shifting towards majority ones, where the presence of bilingualism can decelerate this shift. Typically, bilingualism can sometimes accelerate the reverse shift when the majority language possesses a higher social prestige than the minority one. Our findings emphasize the protective role of bilingualism in mitigating language extinction, particularly when competing languages exhibit distinct social prestige. We expect that our Bayesian computational framework could serve as a useful tool for assessing the roles of socio-linguistic factors in language competition and aiding language preservation and cultural sustainability.

## 1. Introduction

Language is an invaluable asset to humans, whose diversity holds profound importance for both individual speakers and human societies. Extensive research has shown that multilingualism can enhance cognitive executive control and problem-solving skills in the social interactions of individual speakers [1,2], as well as mental flexibility and empathy [3,4]. Beyond acknowledging its transactional nature and role as an instrument for social communication, language also functions as a carrier of the identity, culture and knowledge tradition of a linguistic society [5,6]. Accordingly, preserving the language diversity is pivotal not only for the cultural inheritance but also for social diversity and inclusion, guaranteeing the sustainable development of human cultures. However, a United Nations report from 2022 presents a pessimistic outlook that between 90 and 95% of today’s languages may face extinction or severe endangerment by the end of this century [7]. Recent studies also proposed that the rate of language extinction will increase threefold over the next 40 years, with at least one language disappearing per month [8]. This alarming trend indicates an extensive loss of cultural diversity in the foreseeable future and poses a significant challenge to the sustainable development of human cultures. Consequently, revitalizing endangered languages to preserve them from extinction has now become a universal imperative for humans as a whole.

Language extinction can be brought about by language competition [9], which refers to the dynamics of language usage among interacting individuals who speak different languages [10–14]. It often engenders two primary consequences: language shift and coexistence. Language shift refers to the process by which a population abandons one language in favour of another [15], often accompanied by the loss of an active language tradition and the culture associated with it [16]. This process can be more broadly observed when competing languages carry distinct levels of social prestige owing to their association with different levels of socio-cultural, economic and political power [10,17]. Therefore, language shift commonly leads to the outcome that individual speakers of a minority language in a linguistically and culturally diverse society are inclined to favour the high-prestige language [18]. It has been shown that most recent language extinction events are attributable to language shifts rather than a decline in the population that speaks the language [10]. In contrast to language shift, language coexistence is an ideal scenario in which none of the languages in a particular region faces extinction. In such circumstances, a low-prestige language would survive under pressure from a high-prestige language in a specific community, so that multiple languages could be observed being spoken and used in the region [19]. Besides the language-speaking population, language competition can also trigger contact-induced changes in linguistic structures (e.g. grammar). Previous research has highlighted that the size of second-language learners in a speech community can facilitate the retention or loss of complex linguistic features that are difficult for second-language acquisition [20]. Recently, substantial demographic activities and socio-cultural interactions driven by globalization have induced the frequent occurrence of widespread language competition events, thereby accelerating the extinction of languages [8,21–23]. Therefore, understanding the hidden mechanism of language competition is urgent and essential for slowing down the language shift. Such an understanding would further strengthen linguistic and cultural diversity in addition to facilitating the sustainable development of human cultures.

Language competition is usually investigated on a case-by-case basis in traditional socio-linguistic research [18]. This approach can provide a detailed and comprehensive understanding of the specific socio-cultural, political, economic and historical contexts underlying a particular language competition event [24–26]. Using case-by-case research, sociolinguists have proposed that encouraging bilingualism and enhancing the social prestige of minority languages could be two effective ways to reverse language shifts. For example, Osler [15] pointed out the necessity of fostering a mode of coexistence between minority and dominant languages as an effective means of reversing language shift, suggesting the potential importance of bilinguals and bilingualism in this process. This highlights the complex interplay between bilingualism and language shift within language competition scenarios. On the other hand, measures of language policy and planning may also be effective in preventing the complete loss of an endangered language by elevating its social prestige in a community [27]. In other words, social prestige mediated by policy affects the size of the bilingual population and further changes the dynamics of language competition [28]. However, such an approach is qualitatively case-specific and has limitations in large-scale and cross-context comparisons. As a result, it is challenging to discover the mechanisms underlying language competition globally.

Recent methodological advances in language competition models provide alternative opportunities to address this challenge [29]. These models typically consist of a set of differential equations that can be used to interpret interactions among different language-speaking populations (i.e. monolingual and bilingual groups) and predict their dynamic changes [29]. Modelling studies of language competition primarily began with the early theoretical work of Baggs & Freedman [30,31] and gained momentum with the seminal work of Abrams & Strogatz [32]. The Baggs–Freedman model simulates the interaction between two monolingual groups with the existence of the bilingual group [30,31], while the Abrams–Strogatz model (AS model) investigates language shift between two monolingual groups based on the language social prestige [32]. Various more complex language competition models have been developed based on the AS model by incorporating and parameterizing other socio-linguistic or demographic factors. These factors involve population growth (Kandler’s model) [33], bilingualism (Castello’s model) [34], language similarity (Mira’s model) [35], social structure (Minett’s model) [36] and language diffusion (Zhang’s and Kandler’s models) [33,37]. Nevertheless, the parametric estimations in these models primarily rely on the least squares estimation (LSE). LSE can only produce a single point estimate for each parameter rather than a distribution, thereby failing to quantify its uncertainty or credible range. This limitation forces researchers to assess the effects of socio-linguistic factors solely by simply comparing the differences in their parameter magnitudes. However, without considering uncertainty, it is difficult to distinguish whether their differences result from random noise (e.g. sampling bias) or not, which may raise the misinterpretation of the actual effects of socio-linguistic factors. Accordingly, how to rigorously identify the effects of these socio-linguistic factors, especially bilingualism and social prestige, on shaping language competition remains a critical challenge.

**Figure 1.**
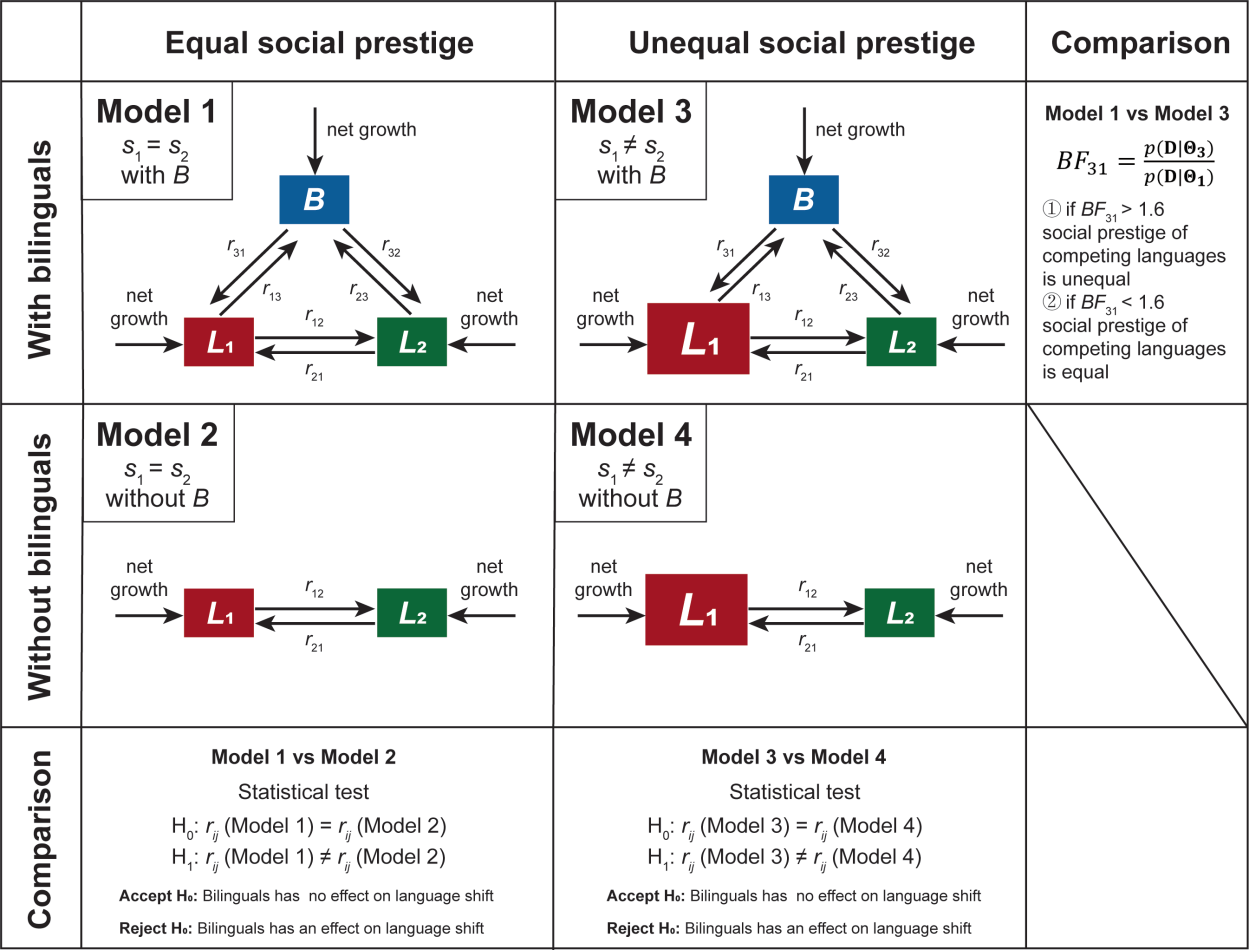
Schematic diagrams for language competition models and evaluation strategy. Model 1 involves equal social prestige and bilinguals. Model 2 involves equal social prestige but no bilinguals. Model 3 involves unequal social prestige and bilinguals. Model 4 involves unequal social prestige but no bilinguals. The comparison between model 1 and model 3 based on the BF aims to assess the equality of social prestige. The statistical comparisons between model 1 and model 2, as well as the ones between model 3 and model 4, aim to assess the role of the bilinguals in language shift. In this study, the statistical comparison is implemented based on the metric of Cohen’s *d*.

Noting this, we here propose a Bayesian parametric estimation framework to quantitatively evaluate the effects of bilinguals and social prestige through rigorous statistical examinations. This framework rests upon model comparisons between baseline and alternative language competition models based on the posterior distributions of their parameters. The baseline model is a Markov process-based language competition model (see details in §4). It demonstrates the most general scenario that any speaker can transit between bilinguals and monolinguals or between different monolinguals, where two competing languages share equal social prestige. In contrast to the baseline model, two alternative models are compared. One excludes bilinguals and the other accounts for disparities in social prestige between two competing languages (see details in §4). Hereby, the equality of social prestige between two competing languages is assessed by comparing language competition models that do or do not involve equal social prestige based on the metric of the Bayes factor (BF) (figure 1; [38]). In addition, the role of bilingualism is evaluated based on comparisons between language competition models that do or do not involve bilinguals based on the metric of Cohen’s *d* (figure 1; [39]). Using the language competition models and Bayesian framework, we then assess the role of bilingualism and social prestige in shaping the competition patterns of six empirical cases.

## 2. Results

To illustrate the generality and universality of our model across different spatiotemporal scales, we collected public data for six empirical competition cases that occurred in different regions (i.e. North America and Europe) and time periods (i.e. century-long and decades-long periods). In North America, these cases were French versus English competition between 1996 and 2017 in Canada (electronic supplementary material, table S1; [40]), English versus French competition between 1996 and 2016 in Montreal (electronic supplementary material, table S2; [40]) and Spanish versus English competition between 1980 and 2010 in the United States (electronic supplementary material, table S3; [41]). In Europe, these cases were the Gaelic-English competition between 1891 and 1971 in Scotland (electronic supplementary material, table S4; [37]), the Welsh-English competition between 1901 and 2001 in Wales (electronic supplementary material, table S5; [37]) and the Catalan-Spanish competition between 2003 and 2018 in Catalonia (electronic supplementary material, table S6; [42]). Each language case encompasses the proportions of two monolingual groups and one bilingual group at a series of time points.

**Figure 2.**
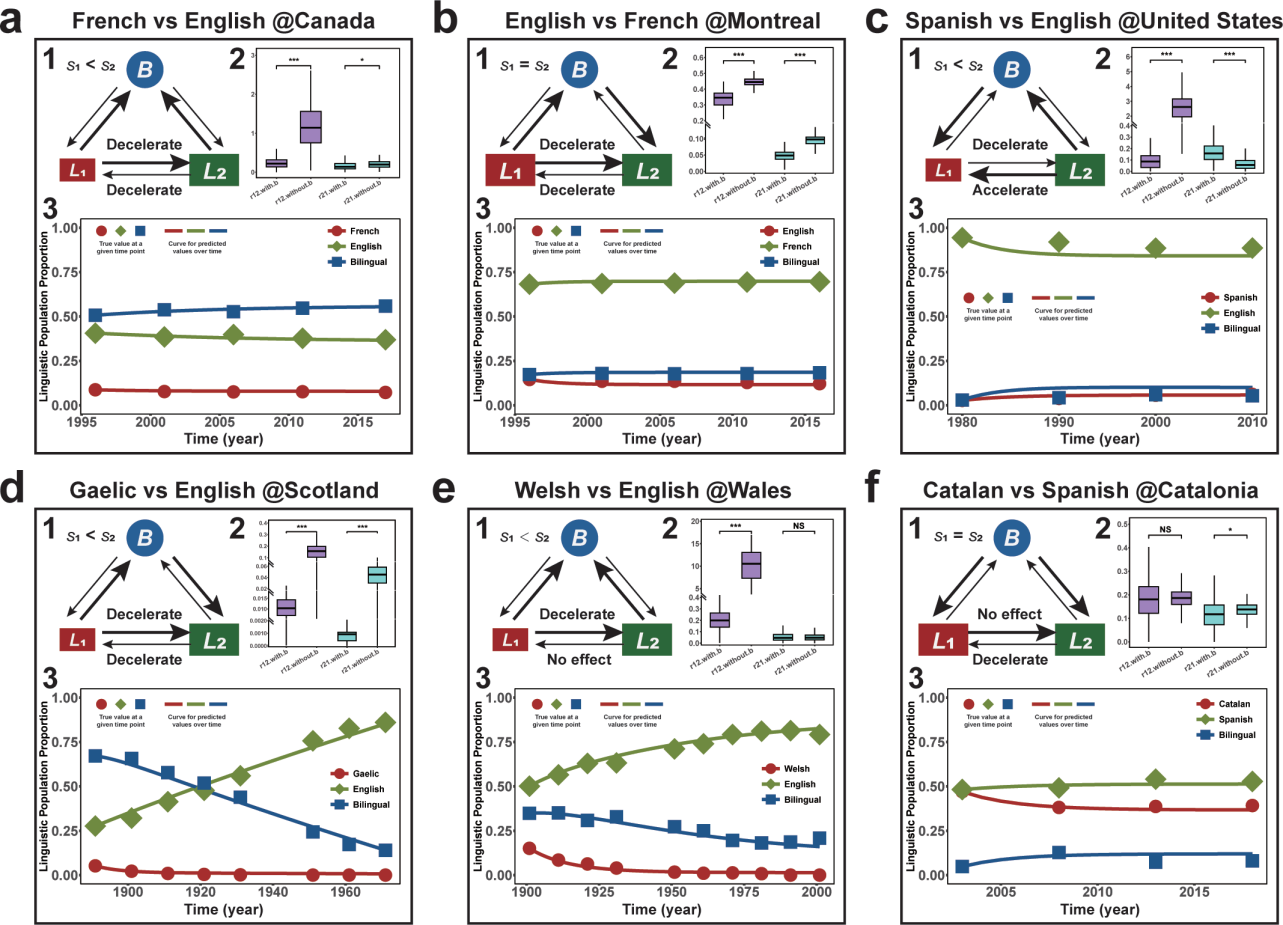
Language competition patterns of six realistic cases with different spatiotemporal scales around the world. Panels (a–f) are composite figures showing the language competition patterns of six empirical cases revealed by our model, each of which encompasses three subfigures labelled 1 to 3. *Subfigure 1* is the network diagram depicting transitions among three linguistic groups. The blue circle denotes the bilingual group, while the red and green rectangles denote the minority **$L_{1}$** and majority **$L_{2}$** monolingual groups. The relative size of the rectangles for **$L_{1}$** and **$L_{2}$** monolingual groups signifies the comparative level of their social prestige. The arrow denotes the transition from one linguistic group to another, with a larger thickness representing a larger transition rate. ‘Accelerate’, ‘decelerate’ or ‘no effect’ indicates the impact of the existence of the bilingual group on the transition rates between two monolingual groups, respectively. *Subfigure 2* is the box plot illustrating the posterior distributions of the transition rates between monolingual groups with and without the existence of the bilingual group. The *y*-axis is inserted breaks for better visualization. The differences among these distributions are measured by Cohen’s *d*. A larger value of Cohen’s *d* indicates a larger difference, which will be annotated by ‘NS’ (0 < *d* < 0.2), ‘*’ (0.2 < *d* < 0.5), ‘**’ (0.5 < *d* < 0.8) and ‘***’ (*d* > 0.8). *Subfigure 3* is the curve plot illustrating the competition dynamics among three linguistic groups. The red, green and blue curves refer to the frequencies of minority **$L_{1}$** monolingual, majority **$L_{2}$** monolingual and bilingual groups predicted by our model over time. The red dot, green rhombus and blue squares refer to the true frequencies of the minority **$L_{1}$** monolingual, majority **$L_{2}$** monolingual and bilingual groups at available time points within the empirical data.

Given that our model is built upon the Bayesian framework, we first determined the optimal and effective prior distributions of the model parameters for each case, according to a series of prior comparisons, prior sensitivity analyses and prior effectiveness validations (see details in the electronic supplementary material, S1, tables S7, S8 and figures S1–S3). With these optimal and effective prior distributions, we then applied our model to investigate the language competition patterns for these six cases (figure 2; table 1).

**Table 1.** Social prestige and role of bilinguals in the language competition cases. (*L*_1_ and *L*_2_ denote the minority and majority languages, respectively.)

competition case: $L_{1}$versus $L_{2}$	equality of social prestige	role of bilinguals		BF
		$L_{1}$→$L_{2}$	$L_{2}$→$L_{1}$	
French versus English (Canada) [40]	≠ ($s_{1}<s_{2}$)	decelerate	decelerate	7.4566
Spanish versus English [41]	≠ ($s_{1}<s_{2}$)	decelerate	accelerate	>1 × 10^3^
Welsh versus English [37]	≠ ($s_{1}<s_{2}$)	decelerate	no effect	2.8438
Gaelic versus English [37]	≠ ($s_{1}<s_{2}$)	decelerate	decelerate	2.7164
English versus French (Montreal) [40]	= ($s_{1}=s_{2}$)	decelerate	decelerate	<2 × 10^−16^
Catalan versus Spanish [42]	= ($s_{1}=s_{2}$)	no effect	decelerate	<2 × 10^−16^

### (a) French versus English competition in Canada

As a result of the colonization of Canada by the French and British starting in the late fifteenth century, both French and English were introduced to this region [43]. Subsequently, competition between these two languages has spanned several centuries, with profound effects on the culture, religion and politics of Canada [43]. Under the Official Languages Act of 1969, English and French hold the same official federal status throughout the nation [44]. Nonetheless, English remains the predominant language in Canada. Based on the available data, we investigated competition between French and English in Canada from 1996 to 2017 (electronic supplementary material, table S1) [40].

As shown in figure 1a, the results of the model comparison revealed that English exhibited more prestige than French in Canada (BF = 7.4566, *s*_2_ = 0.5477 > *s*_1_ = 0.1770, Cohen’s *d* = 1.4871). Moreover, the shift rate from French to English (*r*_12_ = 0.2343) was higher than that from English to French (*r*_21_ = 0.1662). This indicates that in Canada, French monolinguals are more willing to acquire English and become English monolinguals. However, such shift rates between French and English monolinguals would increase in the competition model without bilinguals (*r*_12_ = 1.1560, Cohen’s *d* = 1.7630; *r*_21_ = 0.1987, Cohen’s *d* = 0.2379). This suggests that the presence of bilinguals plays an important role in slowing the shift between the two monolingual groups. This further points to a possibility of decelerating the otherwise more rapid shift from minority French to majority English. All the results are summarized in table 1 and the electronic supplementary material, table S9.

### (b) English versus French competition in Montreal, Canada

To preserve French, the Quebec province enacted the Charter of the French Language in 1977, which made French the exclusive official language within this province [45]. Here, we focused on Montreal, the capital city of Quebec and the second-largest French-speaking city in the world after Paris. We investigated the competition between English and French in Montreal based on the available data from 1996 to 2016 (electronic supplementary material, table S2) [40].

As shown in figure 1b, we did not observe a significant difference between the social prestige of English and French in Montreal (BF < 2 × 10^−16^). Moreover, the rate of English monolinguals shifting to French monolingualism (*r*_12_ = 0.3371) was faster than that of French monolinguals shifting to English monolingualism (*r*_21_ = 0.0475). This indicates that English monolinguals are more willing to acquire French and become French monolinguals in Montreal. Moreover, we found that such rates would increase within the competition model excluding the bilinguals (*r*_12_ = 0.4412, Cohen’s *d* = 1.5687; *r*_21_ = 0.1025, Cohen’s *d* = 1.1704). This suggests that the existence of bilinguals decelerates the shift between French and English monolingualism. All the results are summarized in table 1 and the electronic supplementary material, table S10.

### (c) Spanish versus English competition in the United States

English serves as the predominant language in government and administration throughout the United States and is thus considered the *de facto* national language of sovereignty. However, Spanish is used as an additional language for broadcasting information and providing public services in certain states like New Mexico, Texas and California [41]. With 35 million speakers in the United States, Spanish has stood as the largest minority language in the country, primarily owing to the continuous influx of immigrants from Spanish-speaking countries [46]. We investigated the competition between English and Spanish in the United States based on the available data from 1980 to 2010 (electronic supplementary material, table S3) [41].

As shown in figure 1c, the results of the model comparison (BF > 1 × 10^3^) revealed that English has higher prestige than Spanish in the United States (*s*_2_ = 1.2553 > *s*_1_ = 0.0691, Cohen’s *d* = 4.1873). Nevertheless, the rate of Spanish monolinguals shifting to English monolingualism (*r*_12_ = 0.1067) was lower than that of English monolinguals shifting to Spanish monolingualism (*r*_21_ = 0.1716). This suggests that some policies may have been successfully implemented and encouraged a lot of people to learn Spanish. Moreover, in the competition model without bilinguals, we found that the rate of Spanish monolinguals shifting to English monolingualism would speed up (*r*_12_ = 2.5362, Cohen’s *d* = 2.7731), yet the rate of reversal shift would be slower (*r*_21_ = 0.0647, Cohen’s *d* = 1.0201). This indicates that when competing languages exhibit distinct social prestige, bilinguals can slow the shift from minority to majority languages while sometimes accelerating the shift from majority to minority languages. All the results are summarized in table 1 and the electronic supplementary material, table S11.

### (d) Gaelic versus English competition in Sutherland, Scotland, United Kingdom

Gaelic is an indigenous language in the county of Sutherland in Scotland. However, this language is currently spoken only by a diminishing number of elderly islanders under the strong impact of English [47]. Accordingly, Gaelic is confronted with a severe threat of extinction within decades. Based on the available data, we investigated competition between Gaelic and English from 1891 to 1971 (electronic supplementary material, table S4) [37].

As shown in figure 1d, the results of the model comparison (BF = 2.7164) showed that English exhibited higher prestige than Gaelic in Scotland (*s*_2_ = 0.9655 > *s*_1_ = 0.7911, Cohen’s *d* = 3.2034). We estimated that the language shift was predominantly Gaelic monolinguals shifting to English monolingualism (*r*_12_ = 0.0113 > *r*_21_ = 0.0009). This suggests that Gaelic monolinguals are more willing to use English and become English monolinguals. Moreover, we found that the transition rate between these two monolingual groups increased significantly in the competition model without incorporating the bilinguals (*r*_12_ = 0.1499, Cohen’s *d* = 2.2803; *r*_21_ = 0.0458, Cohen’s *d* = 1.9269). This implies that the presence of bilinguals would suppress the direct shift between monolingual groups, mitigating the minority Gaelic from rapid extinction. All the results are summarized in table 1 and the electronic supplementary material, table S12.

### (e) Welsh versus English competition in Wales, United Kingdom

Welsh used to be the prevalent language in Wales. However, it has gradually become a minority language with the spread of English since the twentieth century [48]. Like other indigenous languages in the United Kingdom, Welsh is in danger of extinction owing to the overwhelming prestige of English. Based on the available data, we investigated competition between Welsh and English from 1901 to 2001 (electronic supplementary material, table S5) [37].

As shown in figure 1e, the result of the model comparison showed that Welsh and English had unequal social prestige in Wales (BF = 2.8438), with English significantly higher than Welsh (*s*_2_ = 0.1939 > *s*_1_ = 0.0130, Cohen’s *d* = 2.5897). Moreover, we observed that the shift between Welsh and English monolingualism was mainly Welsh monolinguals shifting to English monolingualism (*r*_12_ = 0.2137 > *r*_21_ = 0.0599). Moreover, in the competition model excluding the bilinguals, the rate of the shift from Welsh monolinguals to English monolinguals would be higher (*r*_12_ = 10.0877, Cohen’s *d* = 2.8270), yet no significant change was observed regarding the rate of English monolinguals shifting towards Welsh monolingualism (*r*_21_ = 0.0522, Cohen’s *d* = 0.1247). This suggests that bilinguals can slow down the shift from a minority language to a majority language, although they may have no effect on the shift from a majority language to a minority language. All the results are summarized in table 1 and the electronic supplementary material, table S13.

### (f) Catalan versus Spanish competition in Catalonia, Spain

Catalonia is an autonomous community within Spain that acknowledges two official languages: Catalan and Spanish. However, Catalan has a deep-rooted history of political and cultural suppression, which intensified during the military dictatorship of Francisco Franco [49]. Relentless political and societal efforts have led to a revitalization of the Catalan language since the death of Francisco Franco. Using data from the Statistical Yearbook of Catalonia, we investigated the competition between Catalan and Spanish from 2003 to 2018 (electronic supplementary material, table S6) [42].

The result of the model comparison showed that Catalan and Spanish did not exhibit distinct social prestige in Catalonia (BF < 2 × 10^−16^). Moreover, the major shift pattern was Catalan monolinguals shifting to Spanish monolingualism (*r*_12_ = 0.1778 > *r*_21_ = 0.1165). In the competition model without the presence of bilinguals, we observed that the rate of this major shift would not exhibit significant change (*r*_12_ = 0.1850, Cohen’s *d* = 0.0849), although the shift from Spanish monolinguals to Catalan monolingualism would be faster (*r*_21_ = 0.1358, Cohen’s *d* = 0.3007). This result suggests that bilingualism may not play a significant role in the shift from minority Catalonia to majority Spanish. All the results are summarized in table 1 and the electronic supplementary material, table S14.

## 3. Discussion

Modelling language competition contributes to the revitalization and preservation of minority languages, facilitating the sustainable development of the cultures associated with them. In this study, we developed a novel Bayesian computational framework to explore language competition patterns across six empirical cases worldwide. Among these six cases, unequal social prestige between competing languages was identified in four cases, which are French versus English in Canada, Spanish versus English in the United States, Gaelic versus English in Scotland and Welsh versus English in Wales (table 1). By contrast, equal social prestige between competing languages was found in the remaining two cases, which are French versus English in Montreal and Catalan versus Spanish in Catalonia (table 1). Besides, we discovered a general language competition pattern of minority monolinguals shifting towards majority ones. Nevertheless, we found that bilingualism could facilitate the protection of the minority language from two perspectives. Firstly, bilingualism could decelerate the direct shift from the minority monolinguals to the majority ones. It could prevent the rapid loss of minority languages. Secondly, bilingualism can accelerate the direct shift from majority monolinguals to minority ones when the majority language possesses higher social prestige. It could facilitate the growth of the minority monolingual population. In other words, unequal social prestige between competing languages can sometimes invert the role of bilingualism, hastening the shift from the majority to the minority monolingual groups. Consequently, the existence of bilinguals plays an important role in protecting minority languages, especially under conditions of competing languages exhibiting unequal social prestige.

The inequality of language social prestige identified in the competitions of Spanish, French, Gaelic and Welsh against English could be attributed to the hyper-central role of English in the global language system, with its linkage to distinct economic, social and cultural values [50]. Because of these, English holds an unshakeable status in the United States and the United Kingdom, as well as exerting a strong influence across a broader territory of North America, including Canada. Accordingly, Spanish, French, Gaelic and Welsh in these countries still cannot generally attain the social status comparable to English, although some states and regions in these countries have enacted many policies and laws to improve their social status. By contrast, the equality of language social prestige identified in the English versus French competition in Montreal and Catalan versus Spanish in Catalonia could result from the successful implementations of relevant policies and laws, which have produced significant effects for maintaining the minority languages. Specifically, the monolingual official language policy implemented by Montreal effectively elevates the status of French to the extent that it has become almost as prestigious as English in this region [45]. By the same token, robust protective measures have been enacted by Catalonia in education, economy, politics and social–cultural activities [51]. These measures have successfully revitalized Catalan and immensely elevated its social status, which makes Catalan share the same social prestige as Spanish [51].

Although language competition typically results in minority monolinguals shifting to majority ones, bilingualism plays various roles in the preservation of minority languages. Specifically, the presence of bilingualism can decelerate the shift from minority to majority monolingualism, thereby preventing the rapid decline of the minority language. The reason could be that bilinguals could serve as icons to boost the confidence of minority monolinguals to transmit their language to their children. In contrast to the decelerating role, the existence of bilingualism can sometimes accelerate the shift from the majority monolinguals to the minority ones when the majority language exhibits higher social prestige. One plausible explanation is that the significant distinction between the social prestige levels of the competing languages can motivate the government to enact strong protective measures for the low-prestige language and encourage bilingualism. Accordingly, the existence of bilinguals may also create strong incentives for majority monolinguals to learn the minority language or for their children to learn the minority language at school. In other words, the strong social pressure brought forth by language policies, reflected by the existence of bilinguals, can disrupt the existing linguistic hierarchy within a specific region, thus reversing the language shift and leading to the revitalization of endangered languages. Besides, we also find that bilingualism may sometimes have little effect on the direct transition between two monolingual groups, such as in Welsh versus English and Catalan versus Spanish competition. The reason could be that the significant gap between social prestige and the speaker population size of Welsh and English makes the English monolinguals have little motivation to learn Welsh, even when policies and laws support bilingualism. By contrast, the equal social prestige between Catalan and Spanish may diminish the influence of bilingual policies and laws on the inclination of Catalan monolinguals to learn other languages. Nevertheless, we did not observe that the presence of bilingualism would accelerate the shift from minority monolinguals to majority ones. In summary, bilingualism can either positively play a protective role for the minority language or have no significant impact, but it will not contribute to the loss of the minority language.

Our findings favour some existing language competition theories while challenging others. Specifically, they align with Mufwene’s ecological theory [13], which posits that language competition is shaped by prestige and socioeconomic factors. For instance, a language associated with greater power or educational access tends to dominate, potentially driving shifts or extinctions of less-prestige competitors. However, as Mufwene underscores, such outcomes are probabilistic and context-dependent, reflecting adaptation to evolving environments rather than inherent linguistic superiority. Consistent with this view, our models reveal that bilingualism can persist despite prestige imbalances in certain scenarios. This supports Mufwene’s theory that bilingualism mitigates outright dominance and enables feature-level fusion, thereby contributing to preserving minority languages within dynamic ecologies [13]. By contrast, our findings diverge from Fishman’s traditional theory [52]. This theory solely views bilingualism as a transitory stage from minority monolingualism to majority dominance, wherein language unequal prestige disparities will be amplified. In contrast, our observations echoing Mufwene’s theory demonstrate that the role of bilingualism is fluid rather than fixed. Under targeted institutional support and proactive language status planning, its role can be inverted, fostered by shifting ideologies, elevated minority language valuation and broader public-domain functions [53,54]. This observation challenges Fishman’s Graded Intergenerational Disruption Scale, suggesting that reversing language shift deviates from a linear, teleological progression through intergenerational transmission. Instead, it favours a more intricate, cyclical process that may engender novel power dynamics among minority speaker groups [55].

Although our model is also an extension of the AS model, it possesses several notable distinctions from other competition models extended from the AS model, especially Kandler’s model (the detailed comparison with Kandler’s model can be found in the electronic supplementary material, S2 and table S15). These distinctions are primarily manifested in the parametric estimation strategy and model form, which bring some advantages but also limitations to our model. For the parametric estimation strategy, our model is built upon the Bayesian framework, which allows for generating a posterior distribution for each parameter rather than a single value. This enables our model to quantify the uncertainties and credible ranges of model parameters. This further facilitates our model to identify the roles of socio-linguistic factors through rigorous statistical examinations rather than simply comparing the parameter magnitudes as done in Kandler’s model. This reduces the risk of generating misinterpretations of the effects of socio-linguistic factors owing to random noise (e.g. sampling bias). For the model form, our model rests upon ordinary differential equations (ODEs) derived from the Markov process, which assumes constant transition rates among different linguistic groups. Compared to our model, Kandler’s model has a more complex form of partial differential equations (PDEs), which allows the languages to diffuse across space and the transition rates to vary across time. Accordingly, our model has the limitation of only simulating the average language competition pattern at a certain time period, rather than the dynamic competition pattern varying across time and space. Noting this limitation, we further validated the robustness of our model against different time windows by dropping different percentages of the time points in the empirical cases. The results showed that although the concrete estimated values of parameters would exhibit differences, the primary competition patterns identified by our model remained stable within different time windows, particularly the relative size of social prestige between competing languages (the detailed results of these validations are available in the electronic supplementary material, S3 and figures S4, S5).

Despite the limitations of the current version of our model, the advantages of Kandler’s model provide valuable insights for the future improvements of our model from two perspectives. Firstly, we can extend our model from ODE into the PDE framework to simulate the more complex language competition patterns from both spatial and temporal perspectives. Secondly, we can substitute the constant values with functions that can vary across time for transition rates in our model. This can facilitate our model to capture the dynamic competition patterns over time and identify the dynamic effects rather than the average effects of socio-linguistic factors. However, the common limitation of the AS-extended model regarding the definition and estimation of social prestige parameters also warrants further refinement. Specifically, social prestige is an abstract parameter for measuring the social or economic opportunities afforded to the speakers of a certain language. Accordingly, this parameter can only be estimated from the empirical data using model fitting, thereby lacking practical and concrete social and economic meanings. In future studies, we could incorporate empirical social and economic data to aid in the definition and estimation of this parameter. Moreover, other socio-linguistic factors, such as language learning difficulty and structural advantage, can impose pronounced influences on language competition [20], which should be considered in future extensions of our model. Nevertheless, we still hope that our Bayesian language competition model could enrich our understanding of the hidden mechanism of language competition and facilitate the protection and revitalization of minority languages, as well as the cultures associated with them. For instance, it can assist language policymakers in evaluating the effectiveness of bilingual incentive policies by analysing the role of bilingualism in minority language preservation. Similarly, it can help determine whether current policies aimed at enhancing the status of minority languages are effective or require adjustment by examining the equality of language social prestige within different time periods.

## 4. Material and methods

### (a) Language competition data

We collected the time series data of six empirical language competition cases from the public repository. For French versus English (Canada), the data covers the proportions of French and English monolinguals and bilinguals across Canada from 1996 to 2017 with 5-year intervals (electronic supplementary material, table S1; [40]). The data for French versus English (Montreal) similarly tracks these proportions in Montreal from 1996 to 2016 with 5-year intervals (electronic supplementary material, table S2; [40]). For Spanish versus English, the data include the proportions of Spanish and English monolinguals and bilinguals from 1980 to 2010 in the United States, collected at 10-year intervals (electronic supplementary material, table S3; [41]). For Gaelic versus English, the data entail the proportions of Gaelic and English monolinguals and bilinguals from 1891 to 1971 with 10-year intervals in Scotland, United Kingdom (electronic supplementary material, table S4; [37]). For Welsh versus English, the data spans from 1901 to 2001 with 10-year intervals, encompassing the proportions of Welsh and English monolinguals and bilinguals in Wales, United Kingdom (electronic supplementary material, table S5; [37]). For Catalan versus Spanish, the data contain the proportions of Catalan and Spanish monolinguals and bilinguals from 2003 to 2018 in Catalonia, Spain, collected at 5-year intervals (electronic supplementary material, table S6; [42]).

### (b) Language competition model

Equal-prestige model (EPM). The EPM is derived from the continuous-time Markov chain model, which has two types. One type consists of bilinguals, as shown in equation (4.1), while the other does not, as shown in equation (4.2). The detailed descriptions of the parameters and notations are listed in table 2:

**Table 2.** The definitions and units of parameters and notations in our model. (*L*_1_ and *L*_2_ denote the minority and majority languages, respectively.)

	class	symbol	definition	unit
parameter	transition rate	$r_{12}$	transition rate from $L_{1}$ to $L_{2}$ monolingual groups	proportion/year
		$r_{13}$	transition rate from $L_{1}$ monolingual to bilingual groups	
		$r_{21}$	transition rate from $L_{2}$ to $L_{1}$ monolingual groups	
		$r_{23}$	transition rate from $L_{2}$ monolingual group to bilingual group	
		$r_{31}$	transition rate from bilingual group to $L_{1}$ monolingual group	
		$r_{32}$	transition rate from bilingual group to $L_{2}$ monolingual group	
	social prestige	$s_{1}$	social prestige of $L_{1}$ language	no dimension
		$s_{2}$	social prestige of $L_{2}$ language	
notation	linguistics	$M_{1}\left(t\right)$	proportion of $L_{1}$ monolingual group at time t	no dimension
		$M_{2}\left(t\right)$	proportion of $L_{2}$ monolingual group at time t	
		$B\left(t\right)$	proportion of bilingual group at time t	
	demography	N(t)	total population size of monolingual and bilingual groups at time t	person
		$\frac{dN\left(t\right)}{dt}\frac{1}{N\left(t\right)}$	the growth rate for each linguistic group at time t	proportion/year


(4.1) $$\left\{ \begin{array}{l} \frac{dM_{1}\left(t\right)}{dt}=r_{21}M_{2}\left(t\right)+r_{31}B\left(t\right)-\left(r_{12}+r_{13}\right)M_{1}\left(t\right)-\frac{1}{N\left(t\right)}\frac{dN\left(t\right)}{dt}M_{1}\left(t\right)\\ \frac{dM_{2}\left(t\right)}{dt}=r_{12}M_{1}\left(t\right)+r_{32}B\left(t\right)-\left(r_{21}+r_{23}\right)M_{2}\left(t\right)-\frac{1}{N\left(t\right)}\frac{dN\left(t\right)}{dt}M_{2}\left(t\right)\\ \frac{dB\left(t\right)}{dt}=-\left(\frac{dM_{1}\left(t\right)}{dt}+\frac{dM_{2}\left(t\right)}{dt}\right) \end{array}\right.,$$

(4.2) $$\left\{ \begin{array}{l} \frac{dM_{1}^{\prime }\left(t\right)}{dt}=r_{21}M_{2}^{\prime }\left(t\right)-r_{12}M_{1}^{\prime }\left(t\right)-\frac{1}{N\left(t\right)}\frac{dN\left(t\right)}{dt}M_{1}\left(t\right)\\ \frac{dM_{2}^{\prime }\left(t\right)}{dt}=r_{12}M_{1}^{\prime }\left(t\right)-r_{21}M_{2}^{\prime }\left(t\right)-\frac{1}{N\left(t\right)}\frac{dN\left(t\right)}{dt}M_{2}\left(t\right) \end{array}\right..$$

Unequal-prestige model (UPM). The UPM can be regarded as an extension of the EPM model that considers social influence. Like the EPM, the UPM also has two forms. One involves the bilinguals, as shown in equation (4.3), while the other neglects the bilinguals, as shown in equation (4.4). The detailed descriptions of the parameters and notations are listed in table 2:


(4.3) $$\left\{ \begin{array}{l} \frac{dM_{1}\left(t\right)}{dt}=s_{1}r_{21}M_{2}\left(t\right)+s_{1}r_{31}B\left(t\right)-s_{2}\left(r_{12}+r_{13}\right)M_{1}\left(t\right)-\frac{1}{N\left(t\right)}\frac{dN\left(t\right)}{dt}M_{1}\left(t\right)\\ \frac{dM_{2}\left(t\right)}{dt}=s_{2}r_{12}M_{1}\left(t\right)+s_{2}r_{32}B\left(t\right)-s_{1}\left(r_{21}+r_{23}\right)M_{2}\left(t\right)-\frac{1}{N\left(t\right)}\frac{dN\left(t\right)}{dt}M_{2}\left(t\right)\\ \frac{dB\left(t\right)}{dt}=-\left(\frac{dM_{1}\left(t\right)}{dt}+\frac{dM_{2}\left(t\right)}{dt}\right) \end{array}\right.,$$

(4.4) $$\left\{ \begin{array}{l} \frac{dM_{1}^{\prime }\left(t\right)}{dt}=s_{1}r_{21}M_{2}^{\prime }\left(t\right)-s_{2}r_{12}M_{1}^{\prime }\left(t\right)-\frac{1}{N\left(t\right)}\frac{dN\left(t\right)}{dt}M_{1}\left(t\right)\\ \frac{dM_{2}^{\prime }\left(t\right)}{dt}=s_{2}r_{12}M_{1}^{\prime }\left(t\right)-s_{1}r_{21}M_{2}^{\prime }\left(t\right)-\frac{1}{N\left(t\right)}\frac{dN\left(t\right)}{dt}M_{2}\left(t\right) \end{array}\right..$$

In both EPM and UPM models, $M_{1}\left(t\right)$ and $M_{2}\left(t\right)$ denote the proportions of speakers of two monolingual groups of $L_{1}$ and $L_{2}$ languages at time t, respectively. $B\left(t\right)$ denotes the proportions of the bilingual group. $N\left(t\right)$ represents the total number of the bilingual group and two monolingual groups. $s_{1}$ and $s_{2}$ are the social prestige parameters that reflect the economic or social opportunity and status offered to the speakers of $L_{1}$ and $L_{2}$ languages, respectively. A higher value of $s_{i}$ indicates a higher prestige of $L_{i}$ under social pressure. Specifically, if $s_{1}=s_{2}$, the UPM will degenerate as an EPM. In other words, the EPM is the special case of the UPM when $s_{1}=s_{2}$. $M_{1}^{\prime }\left(t\right)=M_{1}\left(t\right)+0.5B\left(t\right)$ and $M_{2}^{\prime }\left(t\right)=M_{2}\left(t\right)+0.5B\left(t\right)$, which satisfy $M_{1}^{\prime }\left(t\right)+M_{2}^{\prime }\left(t\right)=1$.

### (c) Bayesian parametric estimation for the language competition model

To estimate the parameters of the language competition model, we performed Bayesian inference based on the Markov chain Monte Carlo (MCMC) method [56]. Here, we exemplify the estimation procedure using equation (4.3). First, we established the likelihood function for the competition model as shown in equation (4.5). To be specific, we let $F\left(t\right)={\left[M_{1}\left(t\right),M_{2}\left(t\right),B\left(t\right)\right]}^{T}$ be the proportions of the two monolingual groups and a bilingual group estimated by the language competition model at time t. We let $D\left(t\right)={\left[{\tilde{M}}_{1}\left(t\right),{\tilde{M}}_{2}\left(t\right),\tilde{B}\left(t\right)\right]}^{T}$ be empirical proportions of the two monolingual groups and one bilingual group at time t. Accordingly, $D={\left[D\left(t_{1}\right),D\left(t_{2}\right),\ldots ,D\left(t_{n}\right)\right]}^{T}$ and $F={\left[F\left(t_{1}\right),F\left(t_{2}\right),\ldots ,F\left(t_{n}\right)\right]}^{T}$ denote the empirical and estimated proportions of two monolingual groups and bilingual group across time points $t_{1},t_{2},\ldots ,t_{n}$. We assumed that $D\left(t\right)$ follows the matrix normal distribution $MN_{n\times 3}\left(F,E_{n\times n}\sigma_{1}^{2},E_{3\times 3}\sigma_{2}^{2}\right)$, where $\sigma_{i}$ follows the standard normal distribution. Accordingly, the likelihood function of D for the set of all the unknown parameters $\Theta ={\left[r_{ij},s_{1},s_{2},\sigma_{1},\sigma_{2}\right]}_{i,j=1,2,3}$ can be constructed as equation (4.5):


(4.5) $$L\left(D|\Theta \right)=\frac{1}{{\left(2\pi \right)}^{\frac{3n}{2}}\sigma_{1}^{3n}\sigma_{2}^{3n}}exp\left[\frac{-1}{2\sigma_{1}^{2}\sigma_{2}^{2}}{\left(vec(D)-vec(F)\right)}^{T}\left(vec(D)-vec(F)\right)\right].$$

Second, we established the posterior distribution of Θ noted as $p\left(\Theta |D\right)$ based on the Bayesian theorem. Let $p\left(\Theta \right)$ be the prior distribution of Θ. Accordingly, $p\left(\Theta |D\right)$ can be calculated following equation (4.6):


(4.6) $$p\left(\Theta |D\right)=\frac{L\left(D|\Theta \right)p\left(\Theta \right)}{\int L\left(D|\Theta \right)p\left(\Theta \right)d\Theta }.$$

Third, we used the MCMC method to simulate samples of Θ from its posterior distribution $p\left(\Theta |D\right)$. In practice, the settings of prior distributions for six cases are listed in the electronic supplementary material, table S7, which are determined through prior comparisons, prior sensitivity analyses and prior effectiveness validations (see details in the electronic supplementary material, S1, tables S7, S8 and figures S1–S3). Moreover, with the consideration of sampling efficiency, we ran the MCMC with different iterations that can guarantee the convergence of the MCMC for different cases (electronic supplementary material, table S7). The burn-in of the first 60% of samples is set to estimate the mean values and confidence intervals of Θ. F was solved using the *ode* function of the *deSolve* package (1.4.0) in R (4.0.3) [57]. The MCMC method was implemented by the *MCMC* function of the *fmcmc* package (0.5−2) in R (4.0.3) [58].

### (d) Evaluating the equality of social prestige

Evaluating the equality of social pressure was accomplished by comparison between equations (4.1) and (4.3). This comparison was implemented based on the BF [38], which is the most commonly used metric to assess performance among different Bayesian models. To be specific, we let $\Theta_{1}$ and $\Theta_{3}$ be the estimated values of the parameters of equations (4.1) and (4.3), respectively. The BF value between equations (4.1) and (4.3) is calculated as equation (4.7)


(4.7) $${BF}_{31}=\frac{p\left(\Theta_{3}|D\right)p\left(\Theta_{1}\right)}{p\left(\Theta_{1}|D\right)p\left(\Theta_{3}\right)}.$$

According to the criteria proposed by Harold Jeffreys [59], if $BF_{31}<1.6$ then equation (4.3) is not more strongly supported by the data than equation (4.1). In other words, language competition is not significantly affected by social pressure. By contrast, $BF_{31}>1.6$ indicates that equation (4.3) is more strongly supported by the data than equation (4.1). In other words, language competition is significantly influenced by social pressure.

### (e) Identifying the role of bilinguals

The role of bilinguals was identified via a comparison of the transition rate between two monolingual groups. This comparison was performed using Cohen’s *d*, which is a more robust metric that is less sensitive to the sample size than the *p*‐value [39]. The Cohen’s *d* between two vectors, $X_{1}$ and $X_{2}$, was calculated using equation (4.8):


 (4.8) $$Cohen^{\prime }s\;d=\frac{{\bar{X}}_{1}-{\bar{X}}_{2}}{\sqrt{\frac{\left(n_{1}-1\right)sd_{1}^{2}+\left(n_{2}-1\right)sd_{2}^{2}}{n_{1}+n_{2}-2}}}.$$

Here, ${\bar{X}}_{1}$ and ${\bar{X}}_{2}$ are the mean values of $X_{1}$ and $X_{2}$, respectively. $sd_{1}$ and $sd_{2}$ are the standard deviations of $X_{1}$ and $X_{2}$, respectively. $n_{1}$ and $n_{2}$ are the sample sizes of $X_{1}$ and $X_{2}$, respectively. According to Sawilowsky [60], Cohen’s *d* < 0.2 indicates a non-significant difference between $X_{1}$ and $X_{2}$. Cohen’s *d* < 0.5 indicates a small difference between $X_{1}$ and $X_{2}$. Cohen’s *d* < 0.8 indicates a moderate difference between $X_{1}$ and $X_{2}$. Cohen’s *d* > 0.8 indicates a large difference between $X_{1}$ and $X_{2}$.

## Data Availability

The data used in this study are available on OSF [61]. For the convenient application of our approach, all the R codes for our Bayesian language competition models are compressed into an R package, namely ‘LCM’. This R package and its tutorial are also available on OSF [61]. Supplementary material is available online [62].

## References

[1] Bialystok E, Craik FIM, Luk G. 2012 Bilingualism: consequences for mind and brain. Trends Cogn. Sci. **16**, 240–250. (10.1016/j.tics.2012.03.001) 22464592 PMC3322418

[2] Bialystok E, Craik FIM. 2022 How does bilingualism modify cognitive function? Attention to the mechanism. Psychon. Bull. Rev. **29**, 1246–1269. (10.3758/s13423-022-02057-5) 35091993

[3] Greve W, Koch M, Rasche V, Kersten K. 2024 Extending the scope of the ‘cognitive advantage’ hypothesis: multilingual individuals show higher flexibility of goal adjustment. J. Multiling. Multicult. Dev. **45**, 822–838. (10.1080/01434632.2021.1922420)

[4] Dewaele JM, Wei L. 2012 Multilingualism, empathy and multicompetence. Int. J. Multiling. **9**, 352–366. (10.1080/14790718.2012.714380)

[5] Edwards J. 2009 Language and identity: an introduction. Cambridge, UK: Cambridge University Press.

[6] Harrison KD. 2007 When languages die: the extinction of the world’s languages and the erosion of human knowledge. Oxford, UK: Oxford University Press.

[7] UNESCO. 2022 International decade of indigenous languages 2022 – 2032. See https://www.unesco.org/en/decades/indigenous-languages.

[8] Bromham L, Dinnage R, Skirgård H, Ritchie A, Cardillo M, Meakins F, Greenhill S, Hua X. 2022 Global predictors of language endangerment and the future of linguistic diversity. Nat. Ecol. Evol. **6**, 163–173. (10.1038/s41559-021-01604-y) 34916621 PMC8825282

[9] Gao Y, Liu W. 2023 Measures to sustain endangered languages: a bilingual competition model with sliding mode control. PLoS ONE **18**, e0287850. (10.1371/journal.pone.0287850) 37384628 PMC10309631

[10] Kandler A, Unger R. 2018 Modeling language shift. In Diffusive spreading in nature, technology and society (eds A Bunde, J Caro, J Kärger, G Vogl), pp. 351–373. Cham, Switzerland: Springer International Publishing. (10.1007/978-3-319-67798-9_18)

[11] Fill A, Penz H. 2018 The Routledge handbook of ecolinguistics. New York, NY: Routledge.

[12] Mesthrie R, Bradley D. 2018 The dynamics of language: plenary and focus lectures from the 20th international congress of linguists. Cape Town, South Africa: Juta and company (pty) Ltd.

[13] Mufwene SS. 2008 Language evolution: contact, competition and change. London, UK: Bloomsbury Publishing.

[14] Chapel L, Castelló X, Bernard C, Deffuant G, Eguíluz VM, Martin S, Miguel MS. 2010 Viability and resilience of languages in competition. PLoS ONE **5**, e8681. (10.1371/journal.pone.0008681) 20126655 PMC2811194

[15] Ostler N. 2011 Language maintenance, shift, and endangerment. In The Cambridge handbook of sociolinguistics (ed. R Mesthrie), pp. 315–334. (10.1017/cbo9780511997068.024)

[16] Comajoan-Colomé L, Coronel-Molina SM. 2021 What does language revitalisation in the twenty-first century look like? New trends and frameworks, pp. 897–904. Oxford, UK: Taylor & Francis.

[17] Thisanka K, De Silva K. 2023 Can language competition predict finite time extinction of a lower status language? J. Anal. **31**, 1665–1685. (10.1007/s41478-022-00513-y)

[18] Deumert A. 2011 Multilingualism. Cambridge, UK: Cambridge University Press.

[19] McIvor O, McCarty TL. 2017 Indigenous bilingual and revitalization-immersion education in Canada and the USA. In Encyclopedia of language and education:bilingual and multilingual education (eds O García, A Lin, S May), pp. 1–17. Cham, Switzerland: Springer. (10.1007/978-3-319-02324-3_34-1)

[20] Kauhanen H. 2021 A bifurcation threshold for contact-induced language change. Glossa: a journal of general linguistics **7**, 1–32. (10.16995/glossa.8211)

[21] Williams CH. 2002 Book review: Language, economy and society: the changing fortunes of the Welsh language in the twentieth century: J. Aitchison and H. Carter. J. Multiling. Multicult. Dev. **26**, 567–569. (10.1191/0309132502ph388xx)

[22] Ortiz A. 1992 El quechua y el aymara. Madrid, Spain: Colecciones MAPFRE.

[23] Isern N, Fort J. 2014 Language extinction and linguistic fronts. J. R. Soc. Interface **11**, 20140028. (10.1098/rsif.2014.0028) 24598207 PMC3973370

[24] Prēmsīrat S, Hirsh D. 2018 Language revitalization: insights from Thailand. Lausanne, Switzerland: Peter Lang.

[25] Hornberger NH, Coronel-Molina SM. 2004 Quechua language shift, maintenance, and revitalization in the Andes: the case for language planning. Int. J. Soc. Lang. **167**, 9–67. (10.1515/ijsl.2004.025)

[26] Wroblewski M. 2021 Remaking kichwa: language and indigenous pluralism in Amazonian Ecuador. London, UK: Bloomsbury Publishing.

[27] Hornberger NH. 2008 Introduction: can schools save indigenous languages? Policy and practice on four continents. London, UK: Palgrave Macmillan. (10.1057/9780230582491)

[28] McCarty TL. 2018 Revitalizing and sustaining endangered languages. In The Oxford handbook of language policy and planning (eds J Tollefson, M Pérez-Milans), pp. 355–378. Oxford, UK: Oxford Academic.

[29] Boissonneault M, Vogt P. 2021 A systematic and interdisciplinary review of mathematical models of language competition. Humanit. Soc. Sci. Commun. **8**, 1–12. (10.1057/s41599-020-00683-9)38617731

[30] Baggs I, Freedman HI. 1990 A mathematical model for the dynamics of interactions between a unilingual and a bilingual population: persistence versus extinction. J. Math. Sociol. **16**, 51–75.

[31] Baggs I, Freedman HI. 1993 Can the speakers of a dominated language survive as unilinguals?: a mathematical model of bilingualism. Math. Comput. Model. **18**, 9–18. (10.1016/0895-7177(93)90122-F)

[32] Abrams DM, Strogatz SH. 2003 Modelling the dynamics of language death. Nature **424**, 900. (10.1038/424900a) 12931177

[33] Kandler A, Unger R, Steele J. 2010 Language shift, bilingualism and the future of Britain’s Celtic languages. Phil. Trans. R. Soc. B **365**, 3855–3864. (10.1098/rstb.2010.0051) 21041210 PMC2981914

[34] Castelló X, Eguíluz VM, San Miguel M. 2006 Ordering dynamics with two non-excluding options: bilingualism in language competition. New J. Phys. **8**, 308. (10.1088/1367-2630/8/12/308)

[35] Mira J, Paredes Á. 2005 Interlinguistic similarity and language death dynamics. Europhys. Lett. **69**, 1031–1034. (10.1209/epl/i2004-10438-4)

[36] Minett JW, Wang WSY. 2008 Modelling endangered languages: the effects of bilingualism and social structure. Lingua **118**, 19–45. (10.1016/j.lingua.2007.04.001)

[37] Zhang M, Gong T. 2013 Principles of parametric estimation in modeling language competition. Proc. Natl Acad. Sci. USA **110**, 9698–9703. (10.1073/pnas.1303108110) 23716678 PMC3683775

[38] Kass RE, Raftery AE. 1995 Bayes factors. J. Am. Stat. Assoc. **90**, 773–795. (10.1080/01621459.1995.10476572)

[39] Cohen J. 2013 Statistical power analysis for the behavioral sciences. New York, NY: Routledge.

[40] Sutantawibul C, Xiao P, Richie S, Fuentes-Rivero D. 2018 Revisit language modeling competition and extinction: a data-driven validation. J. Appl. Math. Phys. **6**, 1558. (10.4236/jamp.2018.67132)

[41] Templin T. 2019 A language competition model for new minorities. Ration. Soc. **31**, 40–69. (10.1177/1043463118787487)

[42] Statistical institute of Catalonia. Statistical Yearbook of Catalonia. See https://www.idescat.cat/indicadors/?id=aec&n=15042&tema=cultu&lang=en (accessed 17 January 2024).

[43] Burnaby BJ. 2020 Language policy in Canada. See https://www.thecanadianencyclopedia.ca/en/article/language-policy.

[44] Ricento T. 2013 The consequences of official bilingualism on the status and perception of non-official languages in Canada. J. Multiling. Multicult. Dev. **34**, 475–489. (10.1080/01434632.2013.783034)

[45] Kibbee DA. 1998 Language legislation and linguistic rights. In Selected proceedings of the Language Legislation and Linguistic Rights Conference, University of Illinois at Urbana-Champaign, p. 415. Amsterdam, The Netherlands: John Benjamins Publishing. (10.1075/impact.2). http://www.jbe-platform.com/content/books/9789027275073.

[46] Carreira M. 2013 The vitality of Spanish in the United States. Herit. Lang. J. **10**, 396–413. (10.46538/hlj.10.3.10)

[47] ÓhIfearnáin T. 2010 Sociolinguistics of the celtic languages. New York, NY: Routledge.

[48] Durham M, Morris J. 2016 An overview of sociolinguistics in Wales. In Sociolinguistics in Wales (eds M Durham, J Morris), pp. 3–28. London, UK: Palgrave Macmillan. (10.1057/978-1-137-52897-1)

[49] Seoane LF, Loredo X, Monteagudo H, Mira J. 2019 Is the coexistence of Catalan and Spanish possible in Catalonia? Palgrave Commun **5**, 139. (10.1057/s41599-019-0347-1)

[50] DeSwaan A. 2013 Words of the world: the global language system. Hoboken, NJ: John Wiley & Sons.

[51] Strubell M, Boix-Fuster E. 2011 Democratic policies for language revitalisation: the case of Catalan. London, UK: Palgrave Macmillan.

[52] Fishman JA. 2012 Language maintenance, language shift, and reversing language shift. In The handbook of bilingualism and multilingualism (eds T Bhatia, W Ritchie), pp. 466–494. Hoboken, NJ: Blackwell Publishing. (10.1002/9781118332382)

[53] King KA. 2001 Language revitalization processes and prospects: quichua in the Ecuadorian Andes. Bristol, UK: Multilingual Matters.

[54] Kircher R, Kutlu E, Vellinga M. 2024 Promoting minority language use to foster revitalisation: insights from new speakers of West Frisian. Appl. Linguist. **45**, 514–532. (10.1093/applin/amad045)

[55] Hornsby M, McLeod W. 2022 Transmitting minority languages: complementary reversing language shift strategies. London, UK: Springer Nature.

[56] Gilks WR, Richardson S, Spiegelhalter D. 1995 Markov chain Monte Carlo in practice. Boca Raton, FL: CRC Press.

[57] Soetaert K, Petzoldt T, Setzer RW. 2010 Solving differential equations in R : package deSolve. J. Stat. Softw. **33** 1-25. (10.18637/jss.v033.i09)20808728

[58] Yon G, Marjoram P. 2019 fmcmc: a friendly MCMC framework. J. Open Source Softw. **4**, 1427. (10.21105/joss.01427) 35291577 PMC8920406

[59] Jeffreys H. 1998 The theory of probability. Oxford, UK: Oxford University Press.

[60] Sawilowsky SS. 2009 New effect size rules of thumb. J. Mod. Appl. Stat. Methods **8**, 597–599. (10.22237/jmasm/1257035100)

[61] Yang S, Ye T, Bi S, Jin L, Zheng Y, Zhang M. 2025 Data and code from: Bayesian parametric estimation shows the effects of social prestige and bilingualism on reshaping language competition. Open Science Framework Repository. (10.17605/OSF.IO/7DKZJ)41157876

[62] Yang S, Ye T, Bi S, Jin L, Zheng Y, Zhang M. 2025 Supplementary material from: Bayesian parametric estimation shows the effects of social prestige and bilingualism on reshaping language competition. Figshare. (10.6084/m9.figshare.c.8100926)41157876

